# Evaluation of Kidney Function in Cirrhosis: Methods and Pitfalls

**DOI:** 10.3390/diagnostics16132088

**Published:** 2026-07-03

**Authors:** Fernando Gil-Lopez, Namrata Parikh, Martin Mai, Razvan Chirila, Hani M. Wadei

**Affiliations:** 1 Department of Internal Medicine, Mayo Clinic, Jacksonville, FL 32224, USA; gil-lopez.fernando@mayo.edu (F.G.-L.); chirila.razvan@mayo.edu (R.C.); 2 Division of Kidney and Pancreas Transplant, Department of Transplant, Mayo Clinic, Jacksonville, FL 32224, USA; parikhn@upstate.edu (N.P.); mai.martin@mayo.edu (M.M.)

**Keywords:** liver cirrhosis, kidney function, acute kidney injury, chronic kidney disease

## Abstract

Renal dysfunction is highly prevalent among patients with cirrhosis and has major implications for management, prognostication, and liver transplant decision-making. Both acute kidney injury (AKI) and chronic kidney disease (CKD) occur frequently in this population and are driven by a complex interplay of hemodynamic derangements, systemic inflammation, and intrinsic or functional renal injury. Accurate assessment of kidney function is essential, as serum creatinine and estimated glomerular filtration rate (eGFR) calculations directly influence MELD-based transplant allocation, eligibility for simultaneous liver–kidney transplantation (SLKT), and clinical decisions such as drug dosing and peri-transplant immunosuppression strategies. However, creatinine-based assessments are fundamentally limited in cirrhosis due to reduced creatinine production, sarcopenia, expanded volume of distribution, increased tubular secretion, and assay interference from hyperbilirubinemia, resulting in frequent overestimation of true GFR. Alternative methods, including cystatin C-based equations, combined creatinine–cystatin C equations, timed urine clearances, and direct GFR measurement using exogenous filtration markers (iohexol, iothalamate), offer potential advantages but also carry important limitations, particularly in decompensated disease with ascites or inflammation. Newer cirrhosis-specific eGFR equations such as the GRAIL and Royal Free Hospital formulas attempt to address these shortcomings, yet validation studies demonstrate inconsistent accuracy across patient cohorts. This review summarizes the pathophysiologic contributors to renal dysfunction in cirrhosis, evaluates the performance and pitfalls of existing kidney function assessment tools, and highlights the ongoing need for reliable, validated methods tailored to this unique population. Until improved approaches are widely accessible, serum creatinine remains the most practical—albeit imperfect—marker guiding clinical decision-making in cirrhotic patients.

## 1. Introduction

Renal dysfunction is frequently experienced by cirrhotic patients and it is frequently associated with the characteristic abnormal hemodynamic changes specific to cirrhotic patients [1], with or without kidney parenchymal pathology, and can manifest as acute kidney injury (AKI) or chronic kidney disease (CKD) [2,3]. Both AKI and CKD shape long-term outcomes, influence transplant candidacy, and complicate the management of patients with advanced cirrhosis.

AKI is a common complication of decompensated cirrhosis, and its incidence increases as liver disease progress, with almost 50% of cirrhotic patients with ascites developing AKI as a complication at some point [4].

CKD is also becoming increasingly common among cirrhotic patients. According to a recent estimate, the global prevalence of metabolic dysfunction-associated fatty liver disease (MAFLD) is around 30% [5]. In patients with cirrhosis, CKD is associated with worse outcomes for those on the transplant waitlist [6].

Accurately characterizing kidney function in this setting is of central importance in this population for several reasons. Estimated GFR (eGFR) and serum creatinine are not only embedded in prognostic models, inform transplant priority and simultaneous liver–kidney transplant (SLKT) listing, but also guide decisions about appropriate drug dosing [1,7]. Consequently, inaccurate measurement of kidney function can lead to inappropriate drug dosing, under-recognition of high-risk patients, and delayed access to combined organ transplantation. In cirrhotic patients waiting for liver transplantation, renal dysfunction is notably associated with worse outcomes with higher waitlist mortality and inferior post-liver-transplant outcomes [6].

Current methods for evaluating kidney function include serum creatinine- and cystatin C-based estimated glomerular filtration rate (eGFR) equations, timed creatinine clearance, direct glomerular filtration rate (GFR) measurement using exogenous filtration markers as well as a growing number of emerging biomarkers that reflect kidney damage [8,9]. However, none of these is ideal for patients with cirrhosis. In the cirrhotic population, factor such as reduced creatinine production, abnormal tubular creatinine secretion, expanded volume of distribution, hyperbilirubinemia, systemic inflammation, and hemodynamic alterations may lead to significant systematic error in the assessment of kidney function [9,10]. The present review will highlight the importance of accurate estimation of kidney function in cirrhosis, summarize the available methods for estimation of kidney functions and will provide the key advantages, limitations and pitfalls of each method.

## 2. Hemodynamic Abnormalities in Cirrhosis

Patients with cirrhosis experience a series of hemodynamic derangements and neuro-humoral activation that starts early with cirrhosis and progress in parallel to the progression of the liver disease. These abnormalities can culminate into kidney injury.

In cirrhosis, arterial vasodilation is mediated by several potent vasodilators, with nitric oxide (NO) playing the major role [11]. NO production rises in the splanchnic circulation due to shear stress-induced upregulation of endothelial NO synthase (eNOS) and endotoxin-related activation of eNOS [12,13]. Additional mediators, including calcitonin gene-related peptide (CGRP), substance P, carbon monoxide, endocannabinoids, and adrenomedullin, may further contribute to this vasodilated state [14,15,16,17,18]. Marked vascular remodeling develops within the liver and splanchnic circulation, driven by pro-angiogenic factors such as vascular endothelial growth factor (VEGF) and platelet-derived growth factor (PDGF), promoting an extensive network of portosystemic collaterals [19,20].

Portal hypertension drives profound alterations in gut microbiota composition and function, including gut bacterial overgrowth, delayed intestinal transit, and disruption of tight junction proteins. These alterations facilitate translocation of bacteria, bacterial products, and endotoxins from the gut lumen into the splanchnic and then systemic circulation [21].

The inflammatory cascade initiated by bacterial translocation has dual consequences for the kidney: First, pathogen-associated molecular patterns (PAMPs) and proinflammatory cytokines (TNF-α, IL-6) exacerbate splanchnic and systemic vasodilation, compounding the hemodynamic insult and worsening renal hypoperfusion [22]. Second, gut-derived inflammatory mediators cause direct renal injury through microcirculatory dysfunction, microthrombi formation, renal tubular oxidative stress, and tubular damage [22]. In health, the liver acts as a barrier between the gut and the systemic circulation, clearing bacteria and endotoxins via the reticuloendothelial system. Cirrhosis disrupts this protection through both hepatocellular dysfunction and portosystemic shunting, allowing gut-derived products to bypass hepatic clearance [23].

Importantly, disruption of the gut–liver axis confounds kidney function estimation in this population. Gut-derived endotoxemia and systemic inflammation lower creatinine production and render creatinine-based GFR estimates unreliable. Low-grade endotoxemia from increased gut permeability, impaired hepatic lipopolysaccharide clearance, and portosystemic shunting promotes chronic systemic inflammation, which in turn accelerates muscle protein degradation [24,25]. Thus, the gut–liver axis simultaneously drives true kidney injury and influences the biomarkers used to detect it.

Targeting the gut–liver axis represents a potential therapeutic avenue to mitigate kidney injury in cirrhosis. Intestinal decontamination with rifaximin has shown conflicting results: one study in alcohol-related cirrhosis demonstrated improved systemic hemodynamics, reduced endotoxin and cytokine levels, and increased GFR after 4 weeks of treatment [26]. Another retrospective study found that long-term rifaximin use was associated with decreased incidence of AKI and hepatorenal syndrome [27]. However, a randomized, double-blind, placebo-controlled trial found no effect of rifaximin on hepatic venous pressure gradient, systemic hemodynamics, GFR, or vasoactive hormones in patients with decompensated cirrhosis [28]. Fecal microbiota transplantation (FMT) has shown promising early results in patients with cirrhosis [29,30], though its effects on kidney function specifically remain to be established. These therapeutic considerations are relevant to the present review because interventions that modulate the gut–liver axis may alter both true GFR and the biomarkers used to estimate it, further complicating serial kidney function assessment.

The net effect is a fall in systemic vascular resistance and a reduction in effective circulating volume, even though total plasma and blood volumes may be increased. Invasive hemodynamic studies in large cirrhotic cohorts support this physiology, showing low systemic vascular resistance, reduced effective circulating blood volume, and increased splanchnic pooling [31,32].

Reduction in effective arterial blood volume compromises renal perfusion which results in kidney injury [33].

**Figure 1:**
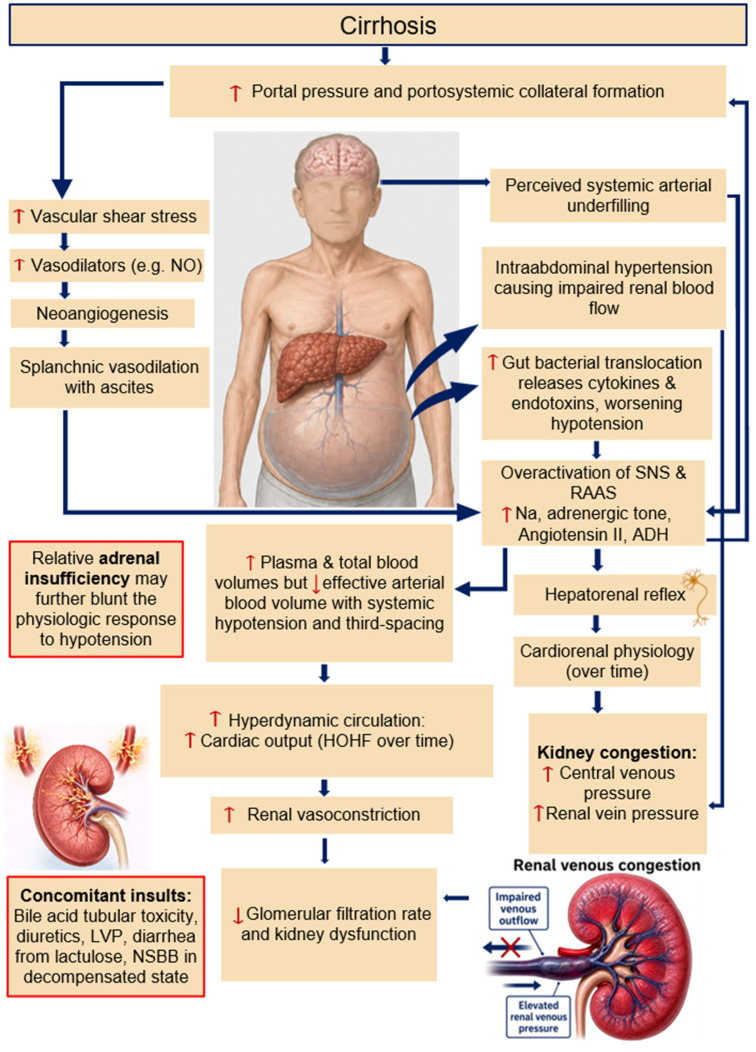
Liver cirrhosis is the initial driver of physiological derangements leading to kidney dysfunction. Central graph depicting cirrhosis with ascites and sarcopenia, sympathetic response-mediated renal vasoconstriction in the left lower quadrant of the graph, and renal venous congestion graph in the right lower corner were created using generative AI (artificial intelligence). The scientific content and directional relationships of the graph were manually created by the authors. Boxes with red margins represent additional contributing factors. Red upward arrows represent an increase in the corresponding factor. Abbreviations: NO, nitric oxide; SNS, sympathetic nervous system; RAAS, renin–angiotensin–aldosterone system; Na, sodium; ADH, antidiuretic hormone; HOHF, high-output heart failure; LVP, large-volume paracentesis; NSBB, nonselective beta-blockers.

Furthermore, as effective arterial volume declines, compensatory systems are triggered: renin–angiotensin–aldosterone system (RAAS) activation, unloading of high-pressure baroreceptors in the carotid body and aortic arch with subsequent sympathetic nervous system (SNS) activation, and non-osmotic antidiuretic hormone (ADH) release. Together, these responses promote intense renal vasoconstriction and a decline in GFR. As cirrhosis worsens, splanchnic vasodilation becomes even more pronounced, perpetuating a vicious cycle of neurohormonal activation and progressive renal vasoconstriction [11]. Figure 1 summarizes the hemodynamic and other systemic derangements that contribute to renal dysfunction in cirrhotic patients.

These derangements give rise to a state of volume overload and ‘third spacing’ of fluid which can dilute traditional markers of kidney function, such as serum creatinine, leading to erroneous determination of both measured and estimated GFR. Moreover, circulating levels of creatinine may be influenced by several factors such as type of assay (false low values in the presence of elevated bilirubin), nutritional status and muscle mass as well as reduced synthesis of precursor creatine in liver. Newer biomarkers such as cystatin C have been proposed to overcome these limitations but their levels can also be influenced by infections and concurrent use of certain medications.

To overcome these limitations, novel biomarkers such as neutrophil gelatinase-associated lipocalin (NGAL), interleukin 18 (IL-18), kidney injury molecule-1 (KIM-1) and liver-type fatty acid-binding protein (L-FABP) have recently generated much interest. However, it should be noted that these are markers of tubular injury and cannot be utilized for estimation of kidney function. Moreover, their levels can also be confounded by infections and inflammation [34].

## 3. AKI and CKD in Cirrhosis: Causes and Implications

### 3.1. Acute Kidney Injury

AKI in cirrhosis is typically classified as prerenal (due to hypoperfusion), intrinsic or parenchymal renal injury, and postrenal obstruction. Prerenal AKI is the most common and often arises from common triggers in this population: aggressive diuretic use, large-volume paracentesis without adequate albumin replacement, diarrhea from lactulose use, and reduced oral fluid intake [11,35]. Hepatorenal syndrome (HRS-AKI, previously called HRS type 1) is a functional form of AKI that typically occurs in the setting of advanced cirrhosis. The defining feature of HRS-AKI is marked renal vasoconstriction, which begins early in the course of liver disease, often months before rise in creatinine becomes clinically apparent [36]. Vasoconstriction progressively intensifies leading to continuous decline in GFR and eventually increase in serum creatinine level and development of AKI [35,37]. Table 1 describes the most common etiologies of AKI in cirrhosis and the key diagnostic features of each one. Desai et al., using a nationally representative dataset, characterized the trends in incidence, healthcare burden and outcomes of hospitalized patients with cirrhosis with and without AKI. In over 3.6 million admissions, 22% had a diagnosis of AKI but this increased from 15% in 2004 to 30% in 2016. This is especially relevant, as AKI admissions are more costly and have a longer length of stay than non-AKI cirrhosis admissions. Additionally, AKI admissions were 3.75 times more likely to result in patient death and were also 3.75 times more likely to result in CKD [38]. A retrospective cohort multicenter study from the HRS-HARMONY consortium analyzed 2063 consecutive hospital admissions in 2019 with cirrhosis and AKI. The most common etiology of AKI was prerenal/hypovolemic AKI (44%), followed by acute tubular necrosis (ATN, 30%) and HRS-AKI (12%). Notably, the 90-day mortality approached 37%, with significant variation by etiology, with the lowest mortality in prerenal AKI (22%), with a substantially higher mortality in HRS-AKI and ATN (49 to 53%). Overall, higher AKI stage and lack of response to therapy were independent predictors of increased mortality, with patients requiring renal replacement therapy having the worse outcomes. Of note, more than one-third of patients at risk developed de novo CKD 3 months after discharge, with similar rates across different etiologies of AKI [39]. Taken together, AKI in patients with cirrhosis carries significant implications, both in terms of economic burden as well as increased risk of subsequent morbidity and mortality.

### 3.2. Chronic Kidney Disease

As mentioned previously, the burden of CKD among patients with cirrhosis has risen in parallel with the growing prevalence of MAFLD and the metabolic syndrome in the general population. These conditions are closely linked to long-standing diabetes mellitus and hypertension, both well-established drivers of chronic kidney disease [40]. MAFLD is increasingly being recognized as a leading indication for liver transplantation (LT), further underscoring the intersection between metabolic liver disease and renal dysfunction [41]. As MAFLD has become more prevalent among LT candidates, the proportion of waitlisted patients with underlying CKD has increased over the past 15 years and now approaches 15% [42]. Although less common than MAFLD in the current era, chronic hepatitis B and C infections remain clinically relevant causes of CKD due to their association with immune-mediated glomerulopathy. Indeed, kidney biopsy studies indicate that nearly two-thirds of LT candidates with otherwise unexplained renal dysfunction have histologic evidence of glomerulonephritis, along with moderate to severe glomerulosclerosis and interstitial fibrosis [43].

Beyond intrinsic kidney disease, portal hypertension itself can contribute to chronic kidney dysfunction, particularly in patients with refractory ascites. Previously termed type 2 HRS, this more indolent form of functional kidney impairment occurs in the absence of structural renal disease and reflects sustained hemodynamic derangements in advanced cirrhosis. Under contemporary definitions, these patients are categorized according to duration as HRS–acute kidney disease (HRS-AKD, <3 months) or HRS–CKD (≥3 months) [44].

Pre-LT CKD has important implications for post-transplant outcomes. In an analysis of 39,719 LT recipients using United Network for Organ Sharing (UNOS) data, pre-LT CKD (GFR < 60 mL/min for ≥90 days or kidney replacement therapy for ≥72 days) was associated with a 16% increased risk of post-LT mortality. Notably, SLKT did not mitigate this risk [42]. These findings underscore the importance of close monitoring of kidney function in cirrhosis, both to identify progressive dysfunction early and to implement strategies that may limit future renal complications.

## 4. The Importance of Accurately Assessing Kidney Function in Cirrhosis

### 4.1. Model for End-Stage Liver Disease (MELD) and Liver Transplant Candidacy

Since 2002, the Model for End-Stage Liver Disease (MELD) was implemented as the main tool to prioritize liver transplantation for liver transplant candidates in the USA. Serum creatinine has been incorporated in the MELD score due to its ability to independently predict short-term mortality in cirrhosis which underscores the pivotal role of kidney dysfunction in prioritizing patients for liver transplantation [45,46].

Accurate estimation of kidney function is also essential for patients undergoing liver transplantation evaluation, as renal dysfunction can markedly influence complications and survival both in the pre- and postoperative periods [47,48].

In 2016, MELD was updated to MELD-Na to incorporate hyponatremia, which is often seen in cirrhotic patients and is also associated with AKI. The intent was to improve the probability of getting transplanted among waitlisted patients [46,49]. However, incorporation of serum creatinine carried a disadvantage for females who have lower skeletal muscle mass and patients with ascites and sarcopenia who also have decreased hepatic creatinine production and poor nutrition [50,51,52]. To mitigate some of these disadvantages, MELD 3.0 was introduced, which incorporated gender and albumin. This has helped to address the disparities that women had experienced with the previous system, marking a step forward in organ allocation [40].

### 4.2. Simultaneous Liver–Kidney Transplantation Allocation Depends on eGFR

The accurate assessment of renal function is central to guide transplantation strategies, both for LT alone and for SLKT, because the Organ Procurement and Transplantation Network (OPTN) criteria depend on GFR/eGFR thresholds sustained over time. Therefore, misclassification can inappropriately drive toward dual-organ listing or, conversely, deny SLKT when it is truly indicated. To standardize the eligibility for SLKT, OPTN established new allocation criteria in 2017 [53]. Under current OPTN guidelines, patients are eligible for SLKT if they have CKD for at least 90 consecutive days as defined by GFR < 60 mL/min and GFR ≤ 30 mL/min at the time of listing or having persistent AKI for at least 6 weeks with either dialysis once every 7 days or GFR < 25 mL/min for 6 consecutive weeks [53].

### 4.3. Adequate Drug Dosing

Many commonly used drugs require adjustment for kidney function, and this is typically obtained with creatinine-based equations. These equations frequently overestimate GFR in patients with cirrhosis and sarcopenia or volume overload, leading to potential drug-related toxicity. By contrast, cystatin C-based equations may underestimate kidney function in this population [8,9]. These implications also extend into the immediate peri-transplant period when selecting the appropriate immunosuppression induction strategy is critical, as renal-sparing approaches such as basiliximab with delayed calcineurin inhibitor initiation, or reduced-dose tacrolimus regimens have been associated with better renal outcomes in liver transplant recipients at higher risk of kidney dysfunction in the post-transplant period [54,55].

### 4.4. Limitations and Complexities of Assessing Renal Function in Cirrhosis

Assessment of renal function in patients with cirrhosis is challenging and carries numerous pitfalls. In the following sections, we will review currently available methods, describing their applications, challenges, and pitfalls.

#### 4.4.1. Measured GFR with Iothalamate or Iohexol Clearance

This method is the current gold standard for measuring the kidney function, also known as measured GFR. This method utilizes the clearance of an exogenous tracer such as iohexol or iothalamate. It yields an absolute, non-body surface area (BSA) indexed value (mL/min) and therefore reflects the individual filtration capacity independent of body-sized indexing, which is especially relevant in patients at the extremes of BSA such as in cirrhosis. This method is also not affected by multiple factors influencing the serum levels of creatinine and cystatin C which are commonly encountered in cirrhotic patients [56].

However, tracer methods are not universally available, have elevated cost, and require specialized workflows. Furthermore, in cirrhotic patients, ascites and associated volume overload can lead to increased volume of distribution, allowing filtration markers to distribute into third space fluid compartments, creating a dilutional effect. As a result, these methods may overestimate GFR in such patients [1,8,57]. A practical way to minimize this error is to estimate clearance after a large-volume paracentesis, to mitigate the effect of third spacing [8,58].

Other direct measurement strategies include isotopic tracers such as 99mTc-DTPA, and 51Cr-EDTA [59,60,61,62]. In practice, these tests are not widely available, and none has been adequately validated in cirrhotic populations for routine GFR determination, although they may still be useful in selected cases [63,64,65,66].

#### 4.4.2. Twenty-Four-Hour Urine for Creatinine and/or Urea Clearance

In routine practice, a 24 h urine collection to calculate creatinine clearance is commonly used as a surrogate for GFR. However, in patients with cirrhosis this approach substantially overestimates true GFR. This discrepancy has long been well known. In a seminal study from 1987 than included 23 patients with cirrhosis with ascites found a major discrepancy between serum creatinine and creatinine clearance, where creatinine often remained “normal” even when creatinine clearance was markedly reduced [64]. This was confirmed in a subsequent meta-analysis including 193 patients with cirrhosis, where measured creatinine clearance substantially overestimated true GFR, surpassing inulin clearance by +13 mL/min/1.73 m^2^, with wide confidence intervals (+60 to −34 mL/min/1.73 m^2^). This was most pronounced in patients with lower GFR with mean overestimation of 18% in those with GFR ≥ 60 mL/min/1.73 m^2^ and 49% in those with GFR < 60 mL/min/1.73 m^2^. Notably, 14% of patients with measured creatinine clearance ≥60 mL/min/1.73 m^2^ had an inulin clearance <30 mL/min/1.73 m^2^, reflecting severe misclassification [65]. This is largely because creatinine is secreted by renal tubules and that secretory component becomes proportionally more pronounced as true GFR falls [8]. Even so, measured creatinine clearance performs better than serum creatinine alone when estimating GFR [64,66]. Measuring urea clearance may help to mitigate some of the issues with creatinine clearance as urea clearance underestimates and creatinine clearance overestimates the true GFR. This composite method which utilizes the mean of both clearances has been proven to outperform estimated GFR using different equations and creatinine clearance alone in patients with GFR < 60 mL/min in patients without cirrhosis [35,67]. However, it suffers from errors in collection and remains vulnerable to shifts in volume status and third spacing, especially because patients with cirrhosis often have frequent bowel movements (or even diarrhea) in the context of lactulose use, making the accurate collection of 24 h urine samples prone to collection error [8].

#### 4.4.3. Serum Creatinine and eGFR Using Creatinine-Based Equations

Equations that calculate GFR are widely employed to estimate renal function in the general population. Depending on the formula, they consider patient characteristics such as age, sex, weight, ethnicity, and laboratory values including serum creatinine, serum urea nitrogen, and serum albumin to generate a predicted GFR. The commonly used Cockcroft-Gault equation, which estimates creatinine clearance and not GFR, has been shown to overestimate GFR in patients with advanced liver disease when compared against inulin clearance (the reference standard), with the greatest overestimation seen when inulin clearance is below 70 mL/min [64,68,69]. Several other equations have also been evaluated in liver transplant candidates. In one report that compared different equations in liver transplant candidates, even the best-performing equation, the model Modification of Diet in Renal Disease-6 (MDRD-6) achieved only 66% of estimates within 30% of GFR measured by I125 iothalamate with lower precision observed in the MDRD-4, MDRD-5 and the Nankivell equations, highlighting clinically significant imprecision at the individual level [68]. The CKD-EPI (Chronic Kidney Disease Collaboration) creatinine equation has generally performed better than MDRD at higher levels of GFR in a review of 12 studies, although most of those studies did not include patients with cirrhosis [70]. However, in cohorts with liver disease CKD-EPI did not consistently outperform MDRD [71,72].

It is important to understand why serum creatinine and creatinine-based eGFR equations which are useful markers of kidney function assessment in the general population, are unreliable in patients with cirrhosis. Serum creatinine is heavily influenced by factors unrelated to glomerular filtration, potentially demonstrating “normal” serum levels even when true GFR is significantly reduced, frequently overestimating kidney function. Moreover, creatine, the precursor of creatinine, is primarily synthesized in the liver. There is good evidence to show that its production is reduced by half in patients with cirrhosis [8,64]. This in turn reduces the production of creatinine in muscles and other tissues. Additionally, sarcopenia and poor nutritional status are common in cirrhosis, further lowering creatinine production [73]. Finally, tubular secretion of creatinine is increased in these patients, particularly in the context of renal dysfunction, which may result in lower serum creatinine concentrations. All these factors contribute to overestimation of GFR in these patients [74,75,76]. Furthermore, some creatinine assays can report falsely low values when bilirubin is markedly elevated or when there are protein-related interferences, although this effect can vary across laboratories [66,77]. Table 2 summarizes the key mechanisms that make serum creatinine and creatinine-based eGFR equations unreliable estimates of true GFR in cirrhotic patients.

Considering all these limitations, serum creatinine can be misleading in cirrhosis. In one report, 37% of patients with cirrhosis with normal creatinine had a measured GFR < 50 mL/min, while another 31% had a GFR between 50 and 80 mL/min [78]. This suggests poor correlation between serum creatinine and true GFR in cirrhosis. The discrepancy is especially pronounced in women, likely due to their lower muscle mass [66,79]. Unfortunately, the discrepancies between estimated and measured GFR have also been illustrated in a large cohort of liver transplant candidates, demonstrating equation-based eGFR often misclassified and overestimated kidney function, which is essential for transplant decisions [80].

#### 4.4.4. Cystatin C and eGFR Using Cystatin C-Based Equations

Cystatin C is produced by all nucleated cells and is cleared primarily by glomerular filtration. Circulating levels are generally not affected by age, sex, muscle mass, and bilirubin [63,79,81,82,83]. Hence, it has been proposed as an alternative marker to serum creatinine, especially in populations with low muscle mass such as cirrhotic patients. Several studies have suggested that cystatin C reflects GFR more accurately than creatinine in patients with cirrhosis, especially in the context of significant GFR decline, and advanced, decompensated cirrhosis [69,82,84,85,86,87,88].

Moreover, cystatin C has been consistently linked to clinically meaningful outcomes, supporting its role beyond a filtration surrogate. In a prospective study by Maiwall et al. [89] cystatin C appeared to better capture risk than creatinine alone, correlating with recurrent AKI and improving mortality prediction when incorporated into MELD components. In patients listed for liver transplant, Mauro et al. reported that elevated cystatin C (≥1.5 mg/L), when considered alongside sarcopenia, identified patients at higher risk for developing acute-on-chronic liver failure (ACLF) and mortality, underscoring its potential value [90]. Similarly, in patients hospitalized with acutely decompensated cirrhosis, Markwardt et al. found that plasma cystatin C predicted subsequent renal dysfunction, ACLF, and 90-day transplant-free mortality, suggesting that cystatin C may reflect integrated systemic risk in addition to kidney filtration [91]. Consistent with this, a multicenter prospective study by Kim et al. showed that cystatin C correlated well with AKI development or progression and with mortality among patients with decompensated cirrhosis, reinforcing its potential utility for short-term risk stratification in hospitalized patients with cirrhosis [92]. Despite its multiple advantages, cystatin C has not been adopted universally to assess kidney function mainly because of practical barriers, such as cost and availability [8]. Importantly, levels can be influenced by factors like infection and by certain medications, such as corticosteroids and calcineurin inhibitors [93]. This is of significant implication for liver transplant recipients.

#### 4.4.5. Estimated GFR Using a Combination of Cystatin C and Creatinine

In cirrhosis, an equation combining creatinine and cystatin C would be especially appealing considering it could potentially “balance out” the limitations that affect each marker [9]. cystatin C-based formulas, including CKD-EPI-Cystatin C and the combined CKD-EPI creatinine and cystatin C equation, may estimate GFR more accurately than creatinine-only equations in cirrhotic patients [9,83,94,95,96,97,98,99,100,101]. Prospective studies in patients with cirrhosis comparing combined creatinine and cystatin C equations against measured GFR demonstrated better performance in contrast to creatinine-only estimates [102]. As mentioned earlier, this is especially relevant in cirrhosis because transplant decisions as well as medication dosing are based on specific thresholds [103]. More recently, race-neutral equations have been adopted in the general population. Although their bias, accuracy, and precision in cirrhosis remain uncertain, a recent study reported lower eGFR values when race-neutral creatinine and cystatin C-based equations were applied and suggested that their use would qualify more patients for simultaneous liver–kidney transplant (SLKT) listing [104].

In summary, within the equation-based methods, the combined creatinine–cystatin C CKD-EPI equation has shown better diagnostic performance than many conventional equations using creatinine or cystatin C alone when compared against measured GFR in cirrhosis, although the accuracy in this population is still significantly affected when compared to its performance in non-cirrhotic individuals and in the setting of ascites and volume overload [8,9,32,58].

#### 4.4.6. Novel Biomarkers for Assessing Kidney Function

Advances in the field of proteomics have led to the emergence of novel biomarkers such as NGAL, IL18, KIM-1, and L-FABP. The use of these biomarkers carries much promise as they permit detection of kidney injury within hours of onset, differentiate prerenal causes of AKI from intrinsic renal injury, and help in prognostication and prediction of long-term outcomes [33].

In a multicenter, prospective cohort study of patients with cirrhosis, analysis of urinary biomarkers of kidney injury such as NGAL, KIM-1, IL18 and L-FABP helped to differentiate between structural and functional causes of AKI (kidney biomarkers and differential diagnosis of patients with cirrhosis and acute kidney injury) [105]. In another study, elevated levels of urinary biomarkers were associated with worse prognosis in patients with cirrhosis and AKI [106]. Elevated urinary levels of these biomarkers may also help to predict short-term mortality, as concluded by a systemic review and meta-analysis of five studies [107].

Of all the biomarkers, NGAL is the most extensively studied in patients with cirrhosis [33]. In a prospective study of 320 consecutive cases of decompensated liver cirrhosis with AKI, urinary NGAL was an independent predictive factor of AKI progression [108].

Although us of these novel biomarkers carries much potential, their use is limited by their lack of widespread availability, validation and standardization. Moreover, studies involving these biomarkers lack homogeneity [109]. Finally, they are markers of tubular injury and provide information about structural kidney damage but not functional capacity. Hence, they have not yet become standard of care [110]. Table 3 depicts a comparison of the performance of creatinine, cystatin C, and novel kidney injury biomarkers across different clinical scenarios in cirrhosis.

#### 4.4.7. Cirrhosis-Specific eGFR Equations

As both creatinine and cystatin C-based equations perform poorly in the presence of ascites and volume overload [8,9], Mindikoglu et al. developed a cirrhosis-specific equation for patients with diuretic refractory ascites and it appeared to outperform CKD-EPI cystatin C and CKD-EPI creatinine–cystatin C in that specific phenotype [98]. However, a more recent study comparing multiple equations head to head reported that even within cirrhosis-specific equations, significant inconsistencies and misclassification occur, with notable discrepancies when different methods were applied to the same patient [57].

In response, two cirrhosis-specific models were developed precisely to reduce the systematic bias that shows up when you apply general population equations to patients with liver dysfunction, ascites, edema, and sarcopenia: the GRAIL (GFR Assessment in Liver Disease) equation and Royal Free Hospital Cirrhosis GFR equation [103,111]. These equations were explicitly developed and tested in the population where serum creatinine correlates poorly with renal function [103,111].

The GRAIL equation was developed with the intention of improving GFR estimation in cirrhotics [103]. The same research group then tested whether substituting serum creatinine with an eGFR obtained using the mentioned equation could improve waitlist mortality risk prediction (MELD-GRAIL-Na model). However, while the MELD-GRAIL-Na model showed better mortality prediction in women and patients with MELD scores above 25, its use has not been validated in other patient groups [112]. Further, it was devised primarily to improve prediction of waitlist mortality in liver transplantation and its use in other aspects of cirrhosis remains unclear. Even in this context, MELD-GRAIL-Na reclassified only about 16% of waitlist patients, suggesting limited impact. Finally, it involves complex calculations which may not be practical.

The Royal Free Hospital (RFH) cirrhosis GFR equation was introduced in 2017 and then validated in an independent cohort as a cirrhosis-specific alternative. Unlike other conventional equations, the RFH model was derived from patients with cirrhosis with a broad spectrum of disease severity (MELD 6–44) and incorporated seven clinical variables (serum creatinine, serum urea, INR, age, serum sodium, sex [male vs. female coefficient], ascites severity [specifically moderate/severe ascites as a coefficient]) [111]. In the initial validation study, the RFH equation demonstrated the highest accuracy for predicting measured GFR, achieving a P30 (precision within 30%) of 89% compared to P30 of 27–75% for MDRD-4, MDRD-6, and CKD-EPI-creatinine. This was consistent across various stages of kidney impairment and liver dysfunction [111].

The RFH score was developed for cirrhosis patients admitted to ICU and may not be applicable to ambulatory patients or patients who are less critically ill [113]. The score also did not account for cardiovascular disease, sarcopenia, frailty or other comorbidities, which limits its prognostic predictiveness. Finally, it performed only marginally better than MELD and could not be validated by other cohorts. In a study that used a Hispanic cohort, the RFH equation outperformed creatinine-only equations. However, its performance was comparable to CKD-EPI-cystatin C. Notably, both RFH and CKD-EPI-cystatin C equations tended to underestimate GFR when it was >60 mL/min/1.73 m^2^ and overestimated it when it was <60 mL/min/1.73 m^2^ [114]. Another retrospective study of 134 cirrhotic patients who had measured GFR by 51Cr-EDTA reported that the RFH equation had significantly lower accuracy than CKD-EPI, MDRD-4, and MDRD-6, highlighting that the performance of the RFH equation may be cohort- and method-dependent and that further prospective validation of this equation is still warranted [115]. A distinctive feature of RFH is that it incorporates the degree of ascites, which addresses a unique feature of cirrhosis but also introduces a subjectivity component that could limit reproducibility [111].

In summary, the GRAIL equation and the RFH score were developed to overcome limitations of creatinine in high-risk subgroups but have limited generalizability, modest incremental benefit, and insufficient external validation. Overall, these models are not yet robust or practical enough to replace conventional approaches in routine clinical practice.

#### 4.4.8. Practical Guide for Clinicians

Until both estimation equations and direct measurement tools are better validated and widely available in cirrhosis, expert opinion continues to support serum creatinine as the most practical marker for the assessment of renal dysfunction, particularly because it is part of the current MELD score and because no alternative strategy has consistently proven to be superior for assessing renal function in this population [116]. Creatinine-based eGFR remains the first-line screening tool but systematically overestimates true GFR due to reduced creatinine production from sarcopenia, malnutrition, and impaired hepatic synthesis—an effect that worsens with advanced disease, female sex, and low GFR. When creatinine is unreliable, serum cystatin C should be added, and the combined CKD-EPI creatinine–cystatin C equation is the least biased estimating equation overall. Measured GFR (using iohexol or iothalamate clearance) is not widely used because of practical difficulties and lack of validation. Cirrhosis-specific scores like GRAIL and RFH may complement standard equations in the transplant setting but lack widespread adoption and have mixed external validation. Finally, novel biomarkers like NGAL and KIM-1 may help in early detection of acute kidney injury, but it should be remembered that they are markers of tubular injury and do not help in determination of GFR. Table 4 includes practical recommendations for the use of eGFR equations and biomarkers for the assessment of kidney function in cirrhosis.

#### 4.4.9. Etiology- and Age-Specific Considerations in Kidney Function Assessment in Cirrhosis

Although the hemodynamic mechanisms underlying renal dysfunction are shared across various etiologies of liver cirrhosis, the spectrum of intrinsic kidney disease and the magnitude of GFR estimation error vary by cause and also by patient age. Alcohol-associated cirrhosis carries a high prevalence of secondary IgA nephropathy and is associated with significant sarcopenia, amplifying creatinine-based GFR overestimation [24,117]. HCV-related cirrhosis is linked to immune complex-mediated membranoproliferative glomerulonephritis, usually in the context of type II mixed cryoglobulinemia, while HBV-related cirrhosis is most commonly associated with membranous nephropathy [118,119].

NAFLD is independently associated with a two-fold increased prevalence of CKD, and the sarcopenic obesity phenotype common in these patients can cause creatinine-based equations to either over- or underestimate GFR depending on the relative contributions of preserved body mass and underlying muscle wasting [120,121].

Patient age introduces additional complexity, as age-related sarcopenia compounds cirrhosis-related muscle loss, further lowering creatinine production and widening the gap between estimated and true GFR. In older patients, cystatin C-based or combined creatinine–cystatin C equations become particularly important, though cystatin C may itself be affected by the higher burden of systemic inflammation in elderly individuals [122]. Conversely, younger patients with NAFLD cirrhosis and sarcopenic obesity may have relatively preserved creatinine levels that mask significant renal impairment. Taken together, these etiology- and age-specific nuances underscore the need for individualized kidney function assessment rather than a uniform approach, although the literature does not yet support distinct GFR estimation algorithms stratified by etiology or age.

## 5. Conclusions

In conclusion, accurate assessment of kidney function in individuals with advanced liver disease remains a critical yet unresolved challenge. Current estimation methods are limited by substantial physiological and methodological constraints, frequently resulting in an imprecise reflection of true renal function. Therefore, continued investigation is needed to develop, refine, and validate more reliable assessment tools that can better support clinical decision-making and improve outcomes in this high-risk population. Meanwhile, expert opinion continues to support serum creatinine as the most practical marker for the assessment of renal dysfunction, particularly due to its incorporation into the MELD score and due to its wide base availability. Future studies should focus on further validation of newer cirrhosis-specific GFR equations and increase the use of isotopic and non-isotopic GFR measurement methods for more accurate GFR assessment and ultimately better care of patients with cirrhosis.

## Figures and Tables

**Table 1:** Different etiologies for AKI in cirrhosis and key diagnostic features. AKI: acute kidney injury; ATN, acute tubular necrosis, SBP: spontaneous bacterial peritonitis; POCUS: point-of-care ultrasound; GI: gastrointestinal; HRS-AKI: hepatorenal syndrome–acute kidney injury; IgA: immunoglobulin A; IVC: inferior vena cava; RAAS; renin–aldosterone–angiotensin system; SNS: sympathetic nervous system, AIN: acute interstitial nephritis; HCV: hepatitis C virus ribonucleic acid; HBV: hepatitis B virus deoxynucleic acid; PCR: polymerase chain reaction; GN: glomerulonephritis.

Etiology	Typical Causes/Pathophysiology	Key Diagnostic Features
**Prerenal AKI (hypoperfusion secondary to low effective arterial volume)**	Very common in cirrhosis; often triggered by decreased oral intake, diuretic use for ascites, large-volume paracentesis, or significant diarrhea from lactulose for encephalopathy.	Clinical history consistent with volume loss, bedside ultrasound/POCUS suggesting low effective circulating volume, bland urinary sediment.
**Ischemic acute tubular necrosis (ATN)**	Usually follows a prolonged prerenal insult; can be precipitated by hypovolemic shock from GI bleeding or distributive shock from infections such as spontaneous bacterial peritonitis (SBP).	History of sustained hypotension or shock, rising creatinine despite volume resuscitation, granular (“muddy brown”) casts on urine microscopy.
**Toxic ATN**	Direct tubular injury from nephrotoxic agents such as vancomycin, aminoglycosides, or fluoroquinolones (e.g., for SBP prophylaxis).	Recent exposure to nephrotoxins, granular casts on urine microscopy, lack of improvement after stopping prerenal triggers.
**Bile cast (cholemic) nephropathy**	Severe cholestasis with intratubular deposition of bile/bilirubin casts in the setting of advanced liver failure.	Markedly elevated serum bilirubin (often >10 mg/dL), bilirubin casts on urine microscopy when present.
**Hepatorenal syndrome-AKI (HRS-AKI)**	Functional AKI driven by severe splanchnic and systemic vasodilation with intense renal vasoconstriction, typically in advanced cirrhosis with ascites, where multiple kidney pathologies may coexist in a single patient, but HRS physiology predominates.	Cirrhosis with ascites and AKI in the absence of shock, recent nephrotoxin exposure, or structural kidney disease (no significant proteinuria, no active urine sediment, normal renal ultrasound). The concept of HRS-AKI as a purely “diagnosis of exclusion” has been abandoned and replaced by a more nuanced and realistic clinical approach that asks whether HRS-AKI is likely to be the predominant driver of kidney dysfunction in a given patient with cirrhosis and ascites.
**Cirrhotic cardiomyopathy-related AKI**	Blunted cardiac contractile reserve and hyperdynamic circulation with RAAS and SNS activation; may limit forward flow to the kidneys in advanced disease.	Echocardiography showing impaired systolic or diastolic function in the appropriate clinical context; often coexists with volume overload. Typically, a contributing factor, rather than a primary, isolated etiology of AKI.
**Abdominal compartment syndrome**	Tense ascites leading to marked intra-abdominal hypertension, renal vein congestion, and reduced renal perfusion.	Sustained intra-abdominal pressure >20 mmHg with evidence of organ dysfunction; tense, painful abdomen; POCUS may show compressed IVC and kidneys.
**Secondary IgA nephropathy**	Cirrhosis-associated immune dysregulation with altered IgA glycosylation and deposition in the glomeruli.	Hematuria and proteinuria on urinalysis; diagnosis confirmed by kidney biopsy when feasible.
**Membranoproliferative GN (often HCV/HBV-related)**	Immune complex-mediated glomerular injury, frequently driven by chronic HCV (often with cryoglobulinemia) or HBV infection.	Hematuria and proteinuria, red blood cell casts on urine microscopy, positive HCV RNA or HBV DNA by PCR; biopsy confirms diagnosis when appropriate.
**Acute interstitial nephritis (AIN)**	Drug-induced hypersensitivity, often from fluoroquinolones used for SBP prophylaxis or proton pump inhibitors used for GI prophylaxis.	Compatible drug exposure history, sterile pyuria, white blood cell casts on urine microscopy; biopsy if diagnosis remains uncertain.
**Obstructive uropathy**	Lower urinary tract obstruction, sometimes precipitated or worsened by medications such as midodrine (via urinary retention) in predisposed patients.	Physical examination, bladder scan, POCUS, and renal ultrasonography showing hydronephrosis or bladder distension.

**Table 2:** Key mechanisms that make creatinine and creatinine-based eGFR unreliable in cirrhosis (reduced creatinine generation, third spacing/dilution, increased tubular secretion, and assay interference), resulting in a creatinine to GFR discordance. Abbreviations: GFR, glomerular filtration rate; eGFR, estimated glomerular filtration rate.

Limitation	Underlying Mechanisms in Cirrhosis	Practical Impact
**Creatinine is unreliable as a filtration marker**	Creatinine is strongly influenced by non-GFR factors in cirrhosis and may remain “normal” even when true GFR is substantially reduced [66,78]; correlation to GFR is poor, often worse in women [79].	Creatinine-based assessments often overestimate kidney function, affecting transplant and perioperative decision-making and drug dosing [66,78].
**Reduced creatine synthesis**	Creatine (creatinine precursor) is primarily synthesized in the liver and is produced at approximately half the rate of healthy controls in cirrhosis [8,64].	Less substrate for creatinine generation implicates lower serum creatinine for a given GFR, leading to overestimation of renal function [8,64].
**Sarcopenia and malnutrition**	Low muscle mass and poor nutritional status are common in cirrhosis and reduce creatinine production [73].	Lower serum creatinine can mask kidney impairment, providing an overestimation of kidney function [66,73,79].
**Third spacing and dilution (ascites/edema)**	Third spacing and redistribution into the peritoneal/interstitial space can lower measured serum creatinine concentrations in advanced disease [66,79].	Dilution can further reduce creatinine levels and cause GFR overestimation, especially in decompensated cirrhosis [66,79].
**Increased tubular creatinine secretion**	Tubular secretion increases, particularly in the setting of renal dysfunction [74,75,76].	Serum creatinine and creatinine-based estimates (and even creatinine clearance) can overestimate true GFR, especially at lower filtration levels [74,75,76].
**Analytical interference (high bilirubin interference)**	Some creatinine assays report falsely low values with marked hyperbilirubinemia or protein-related interferences, and the effect varies by laboratory/platform [66,77].	Inaccurate creatinine values can distort eGFR and staging, particularly in jaundiced patients [66,77].

**Table 3:** Comparison of creatinine, cystatin C and novel biomarkers for assessment of kidney function in patients with cirrhosis. Abbreviations: GFR, glomerular filtration rate; NGAL, neutrophil gelatinase-associated lipocalin; KIM-1, kidney injury molecule-1; AKI, acute kidney injury; MELD, Model for End-Stage Liver Disease; ATN, acute tubular necrosis; HRS-AKI, hepatorenal syndrome–acute kidney injury; HRS, hepatorenal syndrome.

Clinical Scenario	Creatinine	Cystatin C	Novel Biomarkers (NGAL, KIM-1, etc.)
**Compensated cirrhosis**	Overestimates GFR due to decreased creatine production in liver and sarcopenia	Less affected by muscle mass and not affected by creatine production	Not applicable as these are tubular injury markers and cannot estimate GFR.
**Decompensated cirrhosis**	Significantly overestimates GFR due to dilution and third spacing	Performs best as it is not affected by volume status or muscle mass	Not applicable as these are tubular injury markers and cannot estimate GFR.
**Early AKI detection**	Rise in creatinine may be delayed by days—not helpful	Superior to creatinine. MELD–cystatin C improves mortality prediction	NGAL is an independent early predictor of AKI. Other biomarkers like KIM-1 also show similar performance but are less extensively studied.
**Differentiating ATN from HRS-AKI**	Cannot differentiate	Cannot differentiate	NGAL shows excellent performance, but other biomarkers have shown moderate/inconsistent performance.
**Prediction of AKI progression**	Delayed	Improves MELD score accuracy	High levels indicate poor prognosis.
**Prediction of mortality**	Limited performance	MELD–cystatin C superior to MELD–creatinine	Improves MELD prognostic performance.
**Prediction of response to HRS treatment**	Cannot predict	Cannot predict	Urinary NGAL > 220 ng/mL independently associated with non-response.

**Table 4:** Biomarkers and methods to assess kidney function in cirrhosis: A Practical Guide for Clinicians. Abbreviations: eGFR, estimated glomerular filtration rate; CKD-EPI-Cr, Chronic Kidney Disease Epidemiology Collaboration creatinine equation; MDRD, Modification of Diet in Renal Disease; CKD-EPI-CysC, Chronic Kidney Disease Epidemiology Collaboration cystatin C equation; CKD-EPI-Cr-CysC, Chronic Kidney Disease Epidemiology Collaboration creatinine–cystatin C equation; GFR, glomerular filtration rate; mGFR, measured glomerular filtration rate; AKI, acute kidney injury; ACLF, acute-on-chronic liver failure; HRS, hepatorenal syndrome; HRS-AKI, hepatorenal syndrome–acute kidney injury; BUN, blood urea nitrogen; LT, liver transplantation; GRAIL, GFR Assessment in Liver Disease; RFH, Royal Free Hospital; INR, international normalized ratio; NGAL, neutrophil gelatinase-associated lipocalin; ATN, acute tubular necrosis; AUROC, area under the receiver operating characteristic curve; KIM-1, kidney injury molecule-1.

Biomarker/Method	What It Measures	Key Strengths in Cirrhosis	Key Limitations in Cirrhosis	When to Use in Practice
**Creatinine-based eGFR (CKD-EPI-Cr, MDRD)**	Estimated GFR from serum creatinine	Widely available, inexpensive, automated reporting	Overestimates GFR due to reduced creatinine production (muscle wasting, malnutrition, impaired hepatic synthesis)	Routine screening and trending in compensated cirrhosis
**Cystatin C-based eGFR (CKD-EPI-CysC)**	Estimated GFR from serum cystatin C	Not affected by muscle mass or hepatic synthesis; better correlation with mGFR than creatinine-based eGFR; superior prognostic value for AKI, ACLF, HRS, and mortality	Tends to slightly underestimate GFR; may be affected by inflammation, thyroid dysfunction, and corticosteroid use; less widely available	When creatinine-based eGFR is suspected to be inaccurate
**Combined Cr-CysC eGFR (CKD-EPI-Cr-CysC)**	Estimated GFR from both creatinine and cystatin C	Least biased equation overall in cirrhosis population eGFRcys are discordant	Still overestimates GFR at low GFR; performance worse than in non-cirrhotic populations	Preferred equation when both biomarkers are available; especially when eGFRcr and eGFRcys are discordant
**24 h urine creatinine clearance**	Measured creatinine clearance from timed urine collection	Directly measures clearance; does not require specialized reagents	Overestimates GFR due to increased tubular creatinine secretion at low GFR; prone to collection errors; impractical in outpatients and patients with ascites	May be considered when eGFR equations are unreliable and mGFR is unavailable
**Measured GFR (iohexol/iothalamate clearance)**	True GFR via exogenous marker clearance	Gold standard; not affected by endogenous biomarker confounders	Complex, time-consuming, limited availability; confounded by expanded volume of distribution	Pre-transplant evaluation when candidacy is being determined; when eGFR-based decisions have major clinical consequences (e.g., nephrotoxic drug dosing)
**GRAIL (GFR Assessment in Liver Disease)**	eGFR using creatinine, BUN, age, sex, race, albumin; calibrated for liver disease	Better classification of low GFR (30) than CKD-EPI/MDRD before and after LT; predicts CKD development and need for kidney after LT	Poor agreement with mGFR in some validation studies (TDI 82%); not yet widely adopted; requires online calculator	When assessing low GFR (30) in transplant candidates; may complement standard equations for predicting post-LT renal outcomes
**RFH Cirrhosis GFR (Royal Free Hospital)**	eGFR using creatinine, urea, INR, age, sodium, sex, ascites status	Incorporates liver-specific variables (INR, ascites); developed and validated in cirrhosis transplant cohort	Mixed external validation results; one study showed inferior accuracy vs. CKD-EPI/MDRD-6; not widely adopted outside UK	May be useful when liver-specific variables are thought to significantly affect creatinine-based estimates
**Urinary NGAL**	Tubular injury biomarker	Best-studied novel biomarker in cirrhosis–AKI; high accuracy for differentiating ATN from HRS-AKI (AUROC 0.85–0.87); cutoff ~220 ng/mL; predicts terlipressin response and mortality	Not a GFR marker; reflects tubular injury, not filtration; not yet incorporated into guidelines; limited standardization across assays	When AKI develops in decompensated cirrhosis: to differentiate ATN from HRS-AKI; to predict response to terlipressin + albumin; to assess prognosis
**KIM-1**	Tubular injury biomarker upregulated in proximal tubule damage	Marker of structural kidney injury; may complement NGAL	Moderate and inconsistent diagnostic performance across studies; less studied than NGAL in cirrhosis	Investigational; may add value in combination with NGAL and cystatin C for early AKI detection; not recommended as standalone

## Data Availability

The original contributions presented in this study are included in the article. Further inquiries can be directed to the corresponding author.
